# ID 152 - Revisão Sistemática e Metanálise em Rede para Avaliar a Eficácia das Terapias-Alvo Disponíveis no Brasil para o Tratamento da Leucemia Linfocítica Crônica (LLC) Recidivante ou Refratária (R/R)

**DOI:** 10.5327/2237-9622.2025.v34s1.152

**Published:** 2025-11-25

**Authors:** Aline do Nascimento, Isabel Cristina de Almeida Santiago, Rita de Cássia Ribeiro de Albuquerque, Cláudia Lima Vieira, Raphael Duarte Chança, Ricardo Ribeiro Alves Fernandes, Mirian Carvalho de Souza, Laura Augusta Barufaldi

**Keywords:** leucemia linfocítica crônica de células B, recidiva, antineoplásicos, metanálise em rede, revisão sistemática

## Abstract

**Introdução:**

No Brasil, não há diretriz ou protocolo clínico para o tratamento de pacientes com LLC R/R e nem medicamentos incorporados no Sistema Único de Saúde (SUS) para esta população. Com isso, o tratamento da LLC R/R restringe-se ao que se tem disponível para o tratamento de primeira linha, quimioterapia (QT) ou quimioimunoterapia (QIT). Assim, optou-se pela condução de uma revisão sistemática (RS) com metanálise em rede, para avaliar se as terapias-alvo aprovadas no Brasil são mais eficazes do que a QT ou QIT para o tratamento da LLC R/R.

**Método:**

Uma RS de ensaios clínicos randomizados (ECR) com metanálise em rede foi conduzida por dois revisores independentes, conforme as diretrizes metodológicas do Ministério da Saúde e registrada na plataforma PROSPERO (CRD42024516463). Foram feitas buscas nas bases PubMed, Embase, Cochrane e LILACS. Os desfechos de eficácia foram sobrevida livre de progressão (SLP) e sobrevida global (SG). Para avaliação do risco de viés, foi empregada a ferramenta Cochrane risk-of-bias tool for randomized trials (RoB2). Para a metanálise em rede foi utilizada a plataforma NMAstudio, que vincula o pacote "netmeta" do software R e foi adotada a abordagem frequentista para comparar os braços do estudo, incluindo comparações diretas e indiretas, para análise simultânea dos tratamentos.

**Resultados:**

A busca recuperou 1.470 títulos. Foram selecionados ECR que comparavam terapias-alvo (monoterapia ou associadas a QT/QIT/IT) registradas na Agência Nacional e Vigilância Sanitária (Anvisa). Os comparadores poderiam ser esquemas de QT, QIT ou IT, para mimetizar a realidade do SUS. Também foram incluídos ECR que comparavam entre si, terapias-alvo registradas. Com isso, foram elegíveis oito ECR. As intervenções foram: ibrutinibe (IB), ibrutinibe com bendamustina + rituximabe (IB-BR), acalabrutinibe (AC), venetoclax + rituximabe (VEN-R) e zanubrutinibe (ZN). Já os comparadores foram: bendamustina + rituximabe (BR), ofatumumabe (OFT), idelalisibe + rituximabe (ID-R), rituximabe (RIT), ibrutinibe (IB) e ibrutinibe + rituximabe (IB-R). Os desfechos SG e SLP foram em sua maioria classificados com algumas preocupações para risco de viés e apenas um ECR foi considerado com alto risco de viés para SLP. Para análise da rede, o esquema BR foi escolhido como comparador de referência por ser o tratamento mais próximo da prática observada no SUS. Resultados preliminares indicam para SLP maior benefício em relação ao comparador para as tecnologias ZN, VEN-R, IB-R, IB-BR, IB e AC. Destaque para ZN (HR=0,16; IC95% 0,09-0,28), VEN-R (HR=0,19; IC95% 0,14-0,25) e IB-R (HR=0,20; IC95% 0,08-0,51), como os prováveis melhores tratamentos (p-escore 0,92, 0,81 e 0,73, respectivamente). Para SG o maior benefício foi para VEN-R (HR=0,40; IC 95% 0,25-0,63) e IB-R (HR=0,61; IC 95% 0,44-0,83), com p-escore 0,96 e 0,74, respectivamente. As demais comparações não apresentaram diferenças estatisticamente significativas.

**Conclusão:**

A metanálise em rede possibilitou mapear em conjunto os benefícios das tecnologias disponíveis para tratamento da LLC R/R. O ranqueamento obtido para os desfechos de eficácia evidenciam a lacuna no tratamento da LLC R/R ao destacar opções mais eficazes do que o tratamento atualmente ofertado no SUS. Porém, esses resultados devem ser analisados com cautela dado às limitações intrínsecas das comparações indiretas, assim como o número limitado de evidências diretas disponíveis, impossibilitando a avaliação da consistência entre comparações e comprometendo a robustez dos resultados obtidos.

